# Assessing the role of vanadium technologies in decarbonizing hard-to-abate sectors and enabling the energy transition

**DOI:** 10.1016/j.isci.2021.103277

**Published:** 2021-10-13

**Authors:** David A. Santos, Manish K. Dixit, Pranav Pradeep Kumar, Sarbajit Banerjee

**Affiliations:** 1 Department of Chemistry, Texas A&M University, College Station, TX 77843-3255, USA; 2 Department of Materials Science and Engineering, Texas A&M University, College Station, TX 77843-3255, USA; 3 Department of Construction Science, Texas A&M University, College Station, TX 77843-3255, USA; 4 Zachry Department of Civil and Environmental Engineering, Texas A&M University, College Station, TX 77843-3255, USA

**Keywords:** Energy resources, Energy sustainability, Materials science, Materials chemistry, Energy materials

## Abstract

The decarbonization of heavy industry and the emergence of renewable energy technologies are inextricably linked to access to mineral resources. As such, there is an urgent need to develop benchmarked assessments of the role of critical elements in reducing greenhouse gas emissions. Here, we explore the role of vanadium in decarbonizing construction by serving as a microalloying element and enabling the energy transition as the primary component of flow batteries used for grid-level storage. We estimate that vanadium has enabled an avoided environmental burden totaling 185 million metric tons of CO_2_ on an annual basis. A granular analysis estimates savings for China and the European Union at 1.15% and 0.18% of their respective emissions, respectively. Our results highlight the role of critical metals in developing low-carbon infrastructure while underscoring the need for holistic assessments to inform policy interventions that mitigate supply chain risks.

## Highlights

• Enabling the energy transition and deep decarbonization hinges on strategic minerals
• The versatility of vanadium chemistries enables technologies that lower CO_2_ emissions
• In structural applications, vanadium enables a greater economy of materials use
• Vanadium redox flow batteries balance the intermittency of wind and solar power

## Graphical abstract

**Figure:**
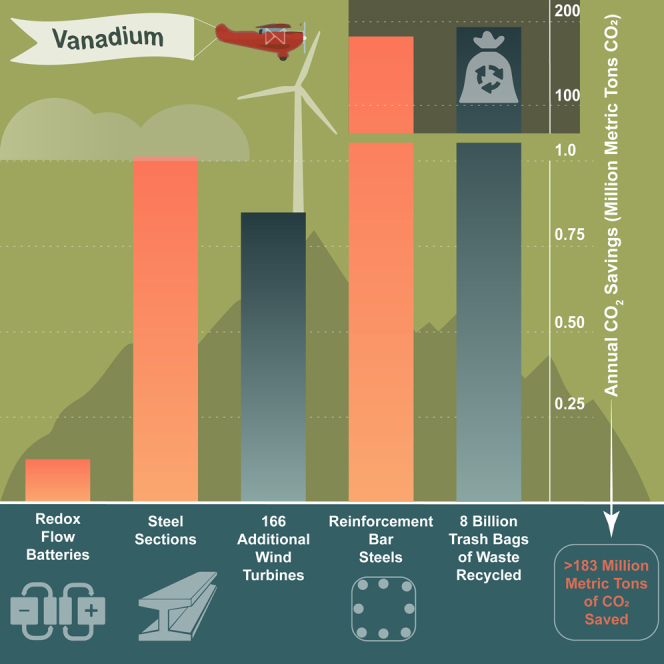

## Introduction

With current economic growth and consumption trends projected to bring about a 4°C rise of the global temperature by 2100, increasingly frequent extreme weather events, a fractious public policy response, and a limited range of negative emission technologies deployable at scale, the world stands at a crossroads with regards to climate change (Millar et al., 2017; Wigley et al., 1981). Maintaining global warming below the ambitious 1.5°C target of the Paris climate agreement will require far-reaching decarbonization (Ramakrishnan et al., 2018). As modern economies brace for an unprecedented energy transition coupled with deep decarbonization of industrial practices, there is considerable concern about the sourcing and utilization of limited natural resources (Child et al., 2018; Graedel et al., 2015). The advent of renewable energy technologies has led to emerging criticality concerns for numerous mineral resources and, in many cases, has stymied their greater market adoption (Hofmann et al., 2018; Sprecher et al., 2017). Indeed, the decarbonization of heavy industry and the emergence of renewable energy technologies are often in direct competition for scarce mineral resources. Benchmarked assessments of critical minerals in reducing the carbon footprint and detailed mapping of materials and energy flows are required to inform public policy design for balancing and expanding access to strategic minerals (Santos et al., 2021). In this article, we seek to develop a longitudinal view of the impact of the transition metal vanadium on the decarbonization of hard-to-abate heavy industries as well as in emerging energy storage applications. The reduction of carbon emissions to achieve global sustainability goals, succinctly denoted as Energiewende in Germany, involves: (1) transitioning from fossil fuels to renewable sources of energy; (2) achieving increased energy efficiencies across the board to reduce overall global energy consumption; and (3) energy storage to decouple electricity generation from use, thus enabling better regulation of supply and demand. The impressive versatility of vanadium redox chemistries enables technologies that transform the manner in which energy is stored and supplied, thus advancing the energy transition by decoupling energy production (from renewable sources) and demand as well as promoting more effective utilization of renewable sources. In addition, the solubility of vanadium and its compounds within iron and titanium-aluminum alloys underpins a greater economy of material use in construction, thereby achieving greater energy efficiency across a traditionally hard-to-abate sector. A combination of policy interventions, technological breakthroughs, and commercial circumstances have led to substantial price fluctuations of this metal in commodity markets, underscoring the need for a holistic industry-wide assessment (Rodby et al., 2020; Santos et al., 2021). Using granular industry-wide materials flow data, we map the use of vanadium in different sectors and develop a rigorous evaluation of its outsized environmental impact across disparate sectors.

**Figure 1:**
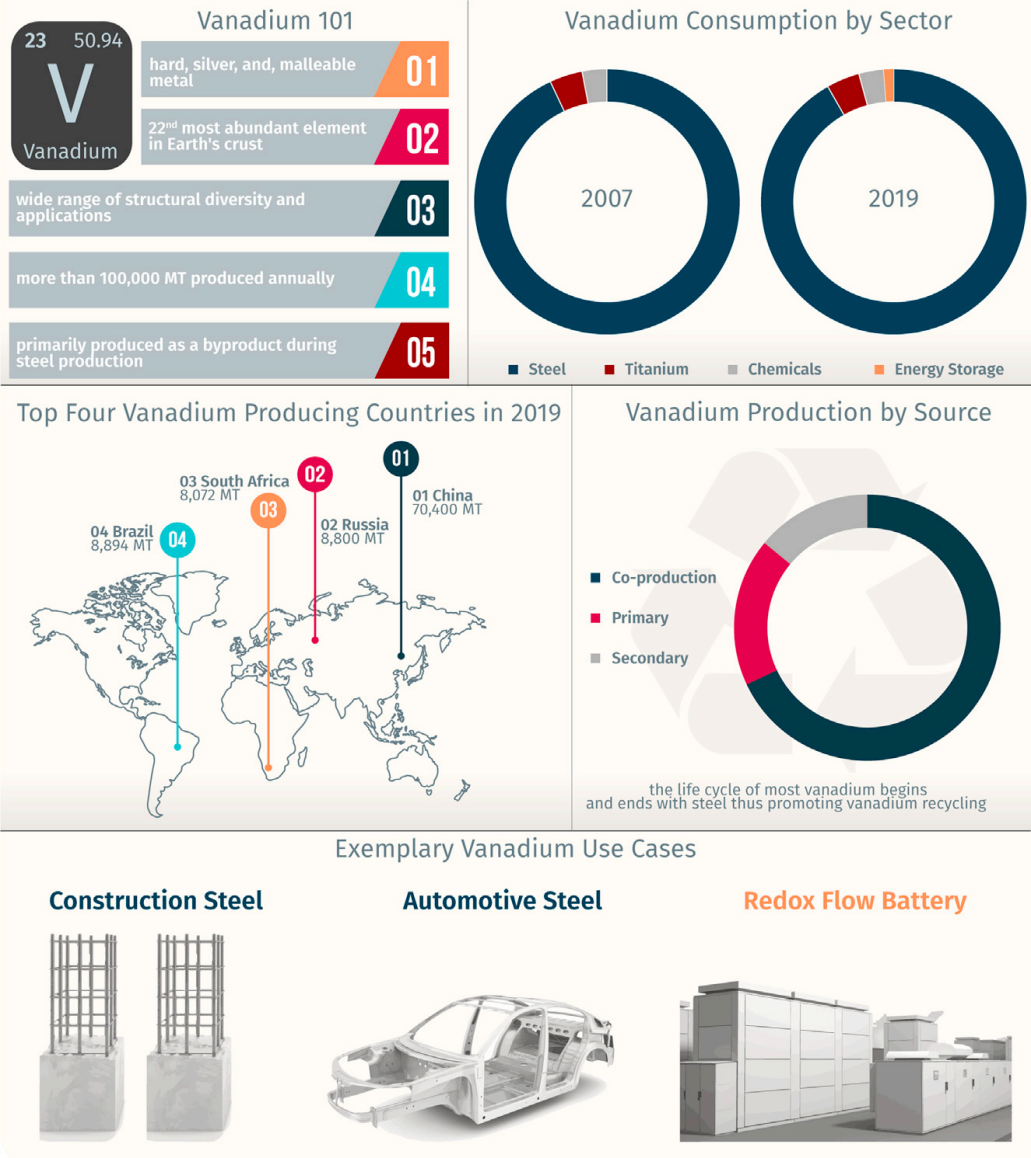
Illustrative overview of the vanadium industry Production and consumption statistics are shown, along with some exemplary use cases featured in this work.

Vanadium (V) is a light, gray, and malleable transition metal found naturally in approximately 65 different minerals. Vanadium’s seven oxidation states from −1 to +5 give rise to an impressive color palette as well as a diverse range of crystal structures of solid-state compounds (De Jesus et al., 2018; White and Levy, 2021). From applications as an additive to strengthen steel (Li and Milbourn, 2013) to the development of neuroemulative circuits that capture the complexity of neurons (Andrews et al., 2019), the versatility of vanadium has led to its application in several industries. Figure 1 illustrates an overview of the vanadium industry and highlights production and consumption statistics obtained from Vanitec, an international trade organization.

Of the approximately 110,000 metric tons (MT) of vanadium produced annually, *ca.* 68% is a co-product formed during steel manufacturing, 18% derives from primary production, and the remaining 14% is from secondary production methods involving the recovery of vanadium from fly ash, petroleum residues, and spent catalysts (Pradeep Kumar et al., 2021; White and Levy, 2021). It is worth noting that secondary production from spent vanadium-bearing refining catalysts is expected to rise sharply as a result of recent regulatory changes issued by the International Maritime Organization (IMO), which limit the maximum sulfur content in bunker fuels (Topali and Psaraftis, 2019). The need for extensive hydrodesulfurization of fractions used in marine fuels will require retooling of refinery processes and more extensive needs for vanadia-based emissions control catalysts. Most of the vanadium produced is consumed by the steel industry as a microalloying agent to strengthen steel. A smaller fraction is diverted to preparing advanced high-strength steel alloys for automotive or cutting tool applications and titanium aluminum alloys for aerospace applications. Notably, the life cycle of the vast majority of vanadium begins and ends with steel, ensuring it is continuously reused and recycled as part of a circular economy (Pradeep Kumar et al., 2021).

With the recent development of vanadium redox flow batteries (VRFB) (Weber et al., 2011), an increasing amount of vanadium has been utilized to create VRFB electrolytes; while the energy storage sector comprised <5% of the vanadium market in 2019, the burgeoning growth of VRFBs and the development of novel V_2_O_5_-based cathodes for intercalation batteries (Andrews et al., 2018; De Jesus et al., 2018; Parija et al., 2016) are anticipated to create a significant rise in market share, potentially disrupting long-established material flows (Rodby et al., 2020). In this work, we highlight use cases of vanadium in three major sectors, i.e., construction (rebar and structural sections), automotive, and energy storage, while emphasizing and quantifying the carbon savings resulting from vanadium-enabled technologies.

The built environment represents a hard-to-abate sector notorious for having a massive carbon footprint, accounting for nearly half of the total global energy consumption (Dixit et al., 2015). The rise of carbon-neutral buildings in the last decade demonstrates the tremendous effort to reduce the operational carbon footprint of buildings through improved energy efficiency and increased reliance on renewable energy sources (Zuo et al., 2012). Unlike operational carbon emissions that can be reduced throughout the use phase by energy-efficient appliances or sustainable practices, the embodied carbon footprint is immutably locked in after the production phase (Akbarnezhad and Xiao, 2017). The manufacturing process of building materials such as steel (Hoffmann et al., 2020) and cement (Monteiro et al., 2017) alone account for *ca.* 16% of the world’s anthropogenic CO_2_ emissions; a deeply embedded dependency on fossil fuels coupled with the energy-intensive production processes and suboptimal process intensification continues to exact a substantial toll on the environment. Mitigating the carbon footprint derived from new construction through greater economy of use of traditional construction materials and design of more sustainable alternatives is imperative to decarbonize the built environment (Bajpayee et al., 2020).

Notably, steel – a mainstay of the construction industry – is already the most recycled material globally; in the United States, approximately 93% of existing steel structures are already comprised of recycled steel, which suggests that the environmental burden avoided by recycling steel is nearing saturation (AISI, 2020). An alternative approach, the use of high-strength low-alloy (HSLA) steels, which offer considerably greater economy of materials use, has gained significant traction due to its ability to reduce the quantity of building materials required to satisfy the performance requirement of a building design (Baker, 2016; Pradeep Kumar et al., 2021;White and Levy, 2021). Carbon emissions are reduced by significantly decreasing the total amount of steel or concrete needed for a structure, i.e., improving material efficiency; the use of HSLA further enables novel architectural designs produced with minimal consumption of steel and other construction materials.

The considerably greater economy of materials use offered by vanadium-alloyed steels has been further utilized by the transportation sector, which accounts for *ca.* 29% of the total US greenhouse gas (GHG) emissions (EPA, 2019). To achieve lower tailpipe emissions, auto manufacturers have introduced novel low-density structural materials (e.g., aluminum and magnesium alloys, carbon fiber composites), or alternatively, advanced high-strength steels that maintain (or improve) functional performance with greater economy of materials use (Geyer, 2008; Taub et al., 2019). However, the carbon-intensive production of many lightweighting materials (up to 20 times greater than steel for aluminum) offsets any savings realized during the use phase from weight reduction (Geyer, 2008; Hickey, 2018; World Auto Steel, 2021). For this reason, applying a life cycle assessment is imperative for an accurate estimation of GHG emissions. In this work, the role of vanadium-based advanced high strength steels (AHSSs) has been discussed qualitatively as an emerging approach for lightweighting in the automotive sector.

In 2019, approximately 63% of electricity generated in the United States was from fossil fuels. In stark contrast, a mere 18% of electricity production originated from renewable energy sources such as wind and solar (EIA, 2019). Despite the seemingly modest contribution from renewable energy sources, the International Energy Agency (IEA) predicts that solar and wind capacity will be doubled globally by 2025, approaching 2,400 gigawatts and exceeding coal and natural gas capacity (IEA, 2020a). Rising solar and wind power generation installations necessitate the development of complementary energy storage technologies, which serve to balance the intermittency of wind and solar power generation. Among candidate energy storage technologies, redox flow batteries have garnered attention on account of their unique ability to scale power and energy density metrics independently to suit application-specific requirements (Weber et al., 2011). In their simplest form, redox flow batteries convert electrical energy into chemical potential energy through reversible electrochemical reactions. Unlike lithium-ion batteries, however, VRFB systems are significantly less susceptible to fire or deflagration risks (Paiss, 2017). Here, the separation of the energy and power components, the use of an aqueous electrolyte, and the comparatively small energy densities gives rise to substantially lower thermal gradients, minimizes available fuel, and essentially eliminates the risk of internal/external shorting (Whitehead et al., 2017). Long cycle life, quick response time, negligible capacity loss over prolonged operation, and excellent safety characteristics have made VRFBs a leading contender in the stationary energy storage segment (Soloveichik, 2015). A distinctive advantage of the VRFB platform is the geographical diversity of vanadium deposits (Imtiaz et al., 2015) and maturity of recycling methods, which mitigates two primary bottlenecks that have significantly impeded the adoption of Li-ion batteries. While vanadium production remains concentrated in China (ca. 63% of total vanadium production), the anticipated demand for vanadium electrolytes has given rise to several announced projects outside of China with a planned production in excess of 20% of the world’s current production volume. Some greenfield and vanadium recovery projects and capacity expansions with a high probability of realization include those from The Ferro-Alloy Resources Group (Kazakhstan), Australian Vanadium Limited (Western Australia), Neometals Limited (Finland), Skåne Vanadium Project (Sweden), Bushveld Minerals (South Africa), and AMG Vanadium (USA and Saudi Arabia). This projected increase in the geographical diversity of vanadium supply has the potential to mitigate many long-term supply risks deeply embedded in the value chain of Li-ion batteries. Several of these projects have been greenlighted upon extensive substantiation of the presence of high-grade alloys. Just as the primary driving force for the transition to renewable energy sources is reducing the carbon footprint, the environmental impact and sustainability of energy storage infrastructure must be considered.

## Results & discussion

### Vanadium construction steels

The composition of HSLA steels is similar to conventional low-carbon steels but includes one or more alloying elements such as vanadium, niobium, or titanium, which significantly increase the yield strength through grain refinement and precipitation strengthening mechanisms (Baker, 2016). Incremental improvements of 15 MPa for every 0.01 weight-percent (wt.%) addition of the microalloying element have been recorded, and yield-strengths as high as 1,000 MPa have been demonstrated for 0.12 wt.% of microalloying (Li and Milbourn, 2013; Pradeep Kumar et al., 2021; Xie et al., 2014). Vanadium has emerged as the primary microalloying element of choice for many HSLA steel producers owing to its superior solubility in the austenite phase, which enables the production of higher strength steels at lower working temperatures (Pradeep Kumar et al., 2021). The primary strengthening mechanisms of vanadium-microalloyed steels include grain refinement and the formation of fine and well-dispersed carbide and nitride precipitates, which offer secondary benefits in the form of protection against corrosion, hydrogen embrittlement, and seismic activity (Baker, 2016; Cho et al., 2018; Hussain et al., 2020; Perrard and Scott, 2007). While alternatives such as niobium provide similar strength benefits, they often necessitate much more energy-intensive thermomechanical treatments, and none match the overall performance of vanadium-based HSLA steels (Kalantar et al., 2019). For two decades, China has been the leading consumer of vanadium, with a greater annual vanadium consumption than the rest of the world combined in recent years. While China exemplifies the massive decarbonization made possible by vanadium alloying, the EU provides a distinct but equally important perspective from an entirely different construction industry wherein the preference for microalloying in construction rebar is partially offset by quenching and self-tempering methods. For this reason, the analysis (*vide infra*) of vanadium-based construction steel considers both EU and China vanadium consumption data to establish a lower and upper bound for estimating global carbon savings in addition to providing insight into regional savings. While the steel assessment outlined in this work focuses on the cradle-to-site gate carbon savings from vanadium microalloying, it is important to note that additional benefits are likely realized during the use phase. Here, resistance to corrosion imbued by vanadium incorporation (the leading cause of deterioration in reinforced concrete structures) (Guzmán and Gálvez, 2017), hydrogen embrittlement (increasingly relevant for ultra-high-strength applications), and seismic activity (of critical importance to earthquake-prone regions) contribute to greater longevity of the built structure, thereby reducing material and energy expenditure during the gate-to-cradle phases.

#### Structural steel

A modeling framework has been developed to account for the carbon benefits of microalloying in steel spanning two levels: component- and building-level analyses. Table S1 shows that at the component level, up to *ca.* 10% and 40% savings in both embodied energy and carbon are calculated for steel beam and columns, respectively, whereas the overall building-level embodied energy and carbon savings from both beams and columns are *ca.* 5%. Lower savings at the building level result from limited choices for section types based on BS4 Part 1 1993 (BS4 Part1, 1993). Conceptually, material savings should increase with increasing tensile strength and thus vanadium addition; in practice, a minimum threshold, determined by the specifications for steel sections, must be met before savings are realized in a practical application (i.e., at the building level). Despite these practical constraints, considerable embodied carbon ends up being saved at the building level due to the vanadium microalloying of steel sections, as discussed in the subsequent sections.

According to the China Iron and Steel Association (CISA), *ca.* 14.5 mMT of structural steel H- and I- sections were produced in 2018. About 69% of this volume was mild steel (Grade Q235), whereas the remainder were high-strength steel sections (>Grade Q345). Therefore, *ca*. 4.48 mMT of high-strength vanadium-microalloyed steel sections were produced in China in 2018. The vanadium content in these sections is 0.035 wt.%, which corresponds to a total of 1,568 MT of vanadium incorporated within steel sections. Using this data, embodied energy and embodied carbon savings are computed and listed in Table 1**.** Vanadium microalloying brings about a 0.29 mMT reduction in steel consumption compared to mild steel (235 MPa). The steel savings, offset by the cost of vanadium incorporation, can be used to quantify embodied energy and embodied carbon savings. An embodied energy savings of 7.33×10^9^ Mega Joules (MJ) and embodied carbon savings of 0.56 mMT are deduced for vanadium-microalloyed 350 MPa steel. The carbon savings correspond to a 0.006% reduction in China’s carbon footprint using China’s fossil fuel-based carbon emissions of 10,000 mMT in 2018 (GCP, 2019).

**Table 1:** Region-specific embodied energy and carbon savings from steel sections

**China**								

Yield strength (MPa)	Steel (MT)	Vanadium (MT)	EE	EC	Savings		China Carbon Savings	
			(×10^9^ MJ)	(mMT CO_2_e)	EE	EC	(mMT CO_2_e)	%
**235**	4,769,896	0	134	10.11	0.00%	0.00%	0.00	0.00%
**350**	4,480,387	1,568	127	9.55	5.47%	5.56%	0.56	0.006%

**European Union (EU-28)**								

Yield strength (MPa)	Steel (MT)	Vanadium (MT)	EE	EC	Savings		EU Carbon Savings	
			(×10^9^ MJ)	(mMT CO_2_e)	EE	EC	(mMT CO_2_e)	%
**235**	1,465,673	0	32	2.24	0.00%	0.00%	0.00	0.00%
**350**	1,376,714	482	30	2.12	5.28%	5.36%	0.12	0.004%

**Rest of world (R.O.W.)**								

Yield strength (MPa)	Steel (MT)	Vanadium (MT)	EE	EC	Savings		R.O.W. Carbon Savings	
			(×10^9^ MJ)	(mMT CO_2_e)	EE	EC	(mMT CO_2_e)	%
**235**	4,320,846	0	121	9.16	0.00%	0.00%	0.00	0.00%
**350**	4,058,591	1,421	115	8.65	5.47%	5.56%	0.51	0.002%

**Global**								

Yield strength (MPa)	Steel (MT)	Vanadium (MT)	EE	EC	Savings		Global Carbon Savings	
			(×10^9^ MJ)	(mMT CO_2_e)	EE	EC	(mMT CO_2_e)	%
**235**	10,556,415	0	286	21.43	0.00%	0.00%	0.00	0.00%
**350**	9,915,692	3,470	271	20.24	5.44%	5.53%	1.18	0.003%

Savings for China, EU-28, Rest-of-world (R.O.W.), and Global regions are shown. The steel column denotes the total steel as per available section steel market data or extrapolated using vanadium usage market data. The EE and EC columns indicate the net savings in embodied energy and carbon for vanadium-microalloyed 350 MPa sections relative to 235 MPa steel. The percentage carbon savings refer to the savings with respect to the regional fossil-fuel-related carbon emissions.

In the European Union (EU-28), *ca.* 9.6 mMT of heavy steel sections were produced in 2018 using 0.05 kg vanadium per ton of steel, i.e., 482 MT of vanadium. Based on an assumption of 0.035% (wt. %) vanadium in steel for 350 MPa sections and a consumption of 482 MT vanadium, the total quantity of vanadium-microalloyed steel is determined to be 1,376,714 MT. A ca. 89,000 MT reduction in steel consumption is obtained by using vanadium-microalloyed steel sections instead of mild steel sections. Table 1 shows the corresponding embodied energy and carbon savings values inferred for the EU-28. Embodied energy savings of 1.66 × 10^9^ MJ and embodied carbon savings of 0.12 mMT are obtained using vanadium-microalloyed 350 MPa steel instead of 235 MPa mild steel. Considering EU-28’s 2018 carbon emissions of 3,400 mMT (GCP, 2019), the carbon savings from the use of vanadium-microalloyed steel sections correspond to a 0.004% reduction in EU-28’s carbon footprint.

In 2018, total global steel section production was 52.5 mMT, with China and EU-28 accounting for combined production of 24.1 mMT; it can be surmised that the rest of the world (R.O.W.) produced 28.4 mMT of steel sections. Considering a vanadium intensity in steel like the EU-28, i.e., 0.05 kg vanadium per ton of steel produced, 1,421 MT of vanadium is incorporated within steel sections. Using an approach analogous to the one applied to the EU-28, i.e., 0.035% (wt. %) vanadium content in section steel and 1,421 MT vanadium yields a total of 4,058,591 MT vanadium-microalloyed steel sections. Table 1 presents embodied energy and carbon savings computed for the R.O.W. Table 1 shows cumulative embodied energy and carbon savings values obtained by adding the relevant metrics for China, EU-28, and R.O.W. A total of *ca.* 0.65 mMT reduction in steel consumption is obtained by replacing mild steel sections with vanadium-microalloyed steel, which corresponds to an embodied energy savings of 15.6 × 10^9^ MJ and embodied carbon savings of 1.18 mMT. Based on the global fossil fuel-based carbon emissions of 36,800 mMT (GCP, 2019) in 2018, the carbon savings translates to a 0.003% reduction of the global carbon footprint.

Embodied energy and carbon savings obtained from the building-level analysis demonstrate the substantial role of vanadium microalloying in reducing steel sections’ environmental impacts and carbon footprint. Impact assessment indicates a 0.004%–0.006% reduction in regional level (EU-28 and China) carbon emissions with respect to their respective 2018 regional fossil carbon footprints. Global analysis shows that vanadium-microalloyed steel sections contribute to an overall CO_2_ savings of 1.18 mMT in 2018, the latest year where an entire set of data is available, which translates to the carbon sequestered by growing nearly 20 million trees for ten years (EPA, 2021).

#### Reinforcement bar steel

Steel reinforcement bars comprise 44% of the total steel used in the construction industry. In a recent publication, the authors of this work performed a detailed life cycle assessment of reinforcement bars using 2017 data and demonstrated the significant role of vanadium microalloying in bringing about embodied energy and carbon savings (Pradeep Kumar et al., 2021). In this article, we calculate the embodied energy and carbon savings using more recent 2019 data—which is particularly instructive given current regulations meant to address seismic resilience that have led to a substantial increase in vanadium consumption. A region-specific analysis is carried out for China and EU-28, and a global scenario is extrapolated using data from China and EU-28 to set upper and lower bounds. Figures S1A and S1B show the proportion of different high strength steel (HSS) rebar grades used and the corresponding vanadium consumption, respectively. The 2019 steel and vanadium data are obtained from China Iron and Steel Research Institute (CISRI) for China. According to CISRI, in China, a total of 227 mMT of HSS rebar was produced in 2019 with 108 mMT of 400 MPa rebar that consumed 32,360 MT of vanadium, 37 mMT of 500 MPa rebar that consumed 22,284 MT of vanadium, and 0.496 mMT of 600 MPa rebar that consumed 496 MT of vanadium. An increase in the production of higher grades such as Grade IV (500 MPa) and Grade V (600 MPa) was observed in 2019, corresponding to stricter implementation and increased compliance with rebar codes put in place after the Sichuan earthquake (Santos et al., 2021). For the EU-28, the steel data is obtained from the World Steel Association and vanadium data from Vanitec. The rebar grade breakdown and corresponding vanadium consumption were obtained using the total rebar steel and vanadium market data and the machine learning model results. As per data obtained from Vanitec, *ca.* 13,000 MT of vanadium was produced in EU-28, and *ca.* 30% of this amount is assumed to be used to produce rebars. In the EU-28, *ca.* 10.6 mMT of rebar was produced in 2019 with *ca.* 9 mMT of 400 MPa rebar consuming 1,183 MT of vanadium and *ca.* 1.5 mMT of 600 MPa rebar that consumed 2,703 MT of vanadium.

**Figure 2:**
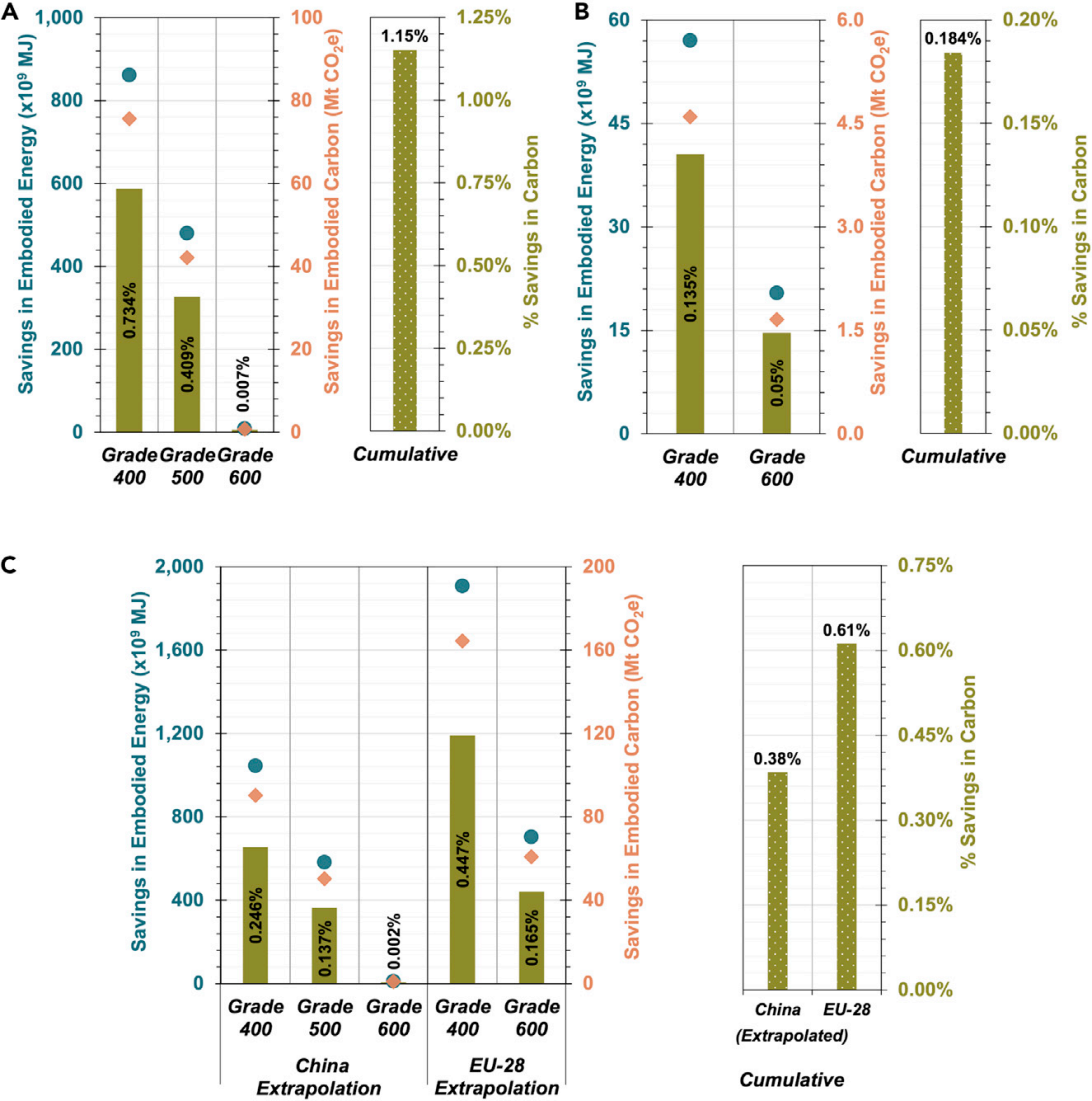
Regional variation in embodied energy and carbon savings (A–C) Savings have been calculated using 2019 data for (A) China; (B) European Union (EU-28); (C). Global (extrapolated to China and EU-28 use scenarios as boundary conditions). Savings in embodied energy (blue markers) and embodied carbon (orange markers) are computed using vanadium in rebar market data and taking vanadium and steel amounts for each steel grade from available regional rebar compositions. For the global scenario analysis, embodied energy and carbon savings are computed using vanadium in rebar market data and vanadium amounts weighted by the proportion of total steel for each grade using China and EU-28 data. The savings in embodied energy and embodied carbon of respective grades are computed with respect to mild steel (250 MPa) as a reference. The % savings in carbon (green bars) are computed with respect to the respective total annual fossil carbon emissions.

Using the most recent (2019) steel and vanadium market data, an LCA is performed as per the procedure delineated in the methodology section. Figure 2 presents a comparison of region-wise and global results obtained using 2019 market data. In China, based on the structural modeling results, *ca.* 39 mMT, 22 mMT, and 0.4 mMT of savings in steel are obtained by replacing mild steel (250 MPa) with grade 400 (400 MPa), grade 500 (500 MPa), and grade 600 (600 MPa), respectively. Figure 2A shows the embodied energy and embodied carbon savings for each rebar grade. Using the 2019 total fossil carbon emission of 10,300 mMT, vanadium microalloying in rebars contribute to 1.15% reduction in the carbon footprint in China. In EU-28, based on the available data and assumptions mentioned above, *ca.* 3.3 mMT and 1.2 mMT of rebar steel savings is observed by replacing 250 MPa steel with 400 MPa and 600 MPa steels, respectively. This directly translates to significant savings in embodied energy and carbon as shown in Figure 2B. A cumulative 0.18% savings is observed in the total carbon emissions in EU-28 with respect to the entire fossil carbon emission of 3,400 mMT in 2019.

At a global level, *ca.* 285 mMT of rebar steel was produced in 2019. We consider two scenarios to bracket use cases. The first scenario involves extrapolation using data from China. A rebar breakdown similar to China is used, which saves *ca.* 49 mMT, 27 mMT, and 0.5 mMT using 400 MPa, 500 MPa, and 600 MPa rebar steel, respectively, instead of mild steel (250 MPa). Figure 2C shows the embodied energy and embodied carbon savings for each rebar grade. Using the total global fossil carbon footprint of 36,800 mMT in 2019, a 0.38% savings in carbon emissions is obtained as a result of replacing mild steel with vanadium-microalloyed rebar steel. Considering the second scenario, EU-28 data is used for the extrapolation and yields *ca.* 88.5 mMT and 33 mMT savings in steel corresponding to mild steel replacement with 400 MPa and 600 MPa rebar steel, respectively. Considerable savings in embodied energy and carbon are obtained using this scenario, as shown in Figure 2C. The carbon savings translate to a cumulative 0.61% savings in carbon footprint globally using the 2019 total global fossil carbon emissions. Cumulatively, using the China and EU-28 extrapolation, a 0.38–0.61% reduction of the total global carbon emissions is estimated, demonstrating the considerable significance of vanadium as a microalloying material in improving the sustainability of the construction industry.

Based on this analysis, vanadium-microalloyed reinforcement bars clearly offer significant embodied energy and carbon benefits. We estimate that between 141.7 and 225.2 mMT of CO_2_ (based on the China and EU-28 extrapolations, respectively) was saved worldwide in 2019 due to vanadium-microalloyed reinforcement bars in construction. Here, the average savings between the lower and upper bounds (183.5 mMT of CO_2_) equates to the expected quantity of carbon sequestered by as much as three billion trees grown for ten years (EPA, 2021). Region-specific analyses performed for China and the EU-28, likely capture the upper and lower bounds of expected savings – given the limited availability of data from the EU-28, the upper bounds of this analysis are likely subject to greater uncertainties.

### Vanadium automotive steels

The use of advanced high-strength steel (AHSS) has rapidly increased in automotive applications due to these alloys’ superior structural properties and unique ability to lower carbon emissions across all phases of the automotive life cycle (Keeler et al., 2017; Lesch et al., 2017). Much like the HSLA steels utilized in construction, AHSS offers a considerably greater economy of materials use relative to baseline steels by meeting the performance requirements of a component with considerably less material, thereby affording weight savings and increased fuel economy. Unlike HLSA steels, the functional performance of AHSS is engineered both from alloying and precise thermomechanical processing, which work in tandem to achieve a multiphase texture characterized by a combination of strength and ductility not attainable with conventional HSLA steels (Keeler et al., 2017; Taub et al., 2019). Some examples of AHSS include Dual Phase (DP), Complex-Phase (CP), Ferritic-Bainitic (FB), Martensitic (MS), Transformation-Induced Plasticity (TRIP), Hot-Formed (HF), and Twinning-Induced Plasticity (TWIP) steels. As a result of the remarkable efficiency of microalloying elements such as vanadium (typically comprising less than 0.5 wt.%) to affect large increases in strength, the carbon-footprint of AHSS production is comparable to that of conventional steel, the most widely recycled material, and many times less than that of aluminum, magnesium, and carbon fiber composites (Broadbent, 2016; Geyer, 2008). An extensive report by the Steel Recycling Institute (SRI) demonstrates savings in mass and carbon from an AHSS-intensive body in white (BIW) over the baseline comparison in 100% of the 5,000 case studies and more than 90% of cases when compared to aluminum-intensive designs (Sebastian and Thimons, 2017). Similar findings by Tata Steel suggest up to 30.5% savings in the total lifecycle carbon footprint from an AHSS-intensive design over an aluminum-intensive design, and up to a 34.8% decrease relative to a fiber-glass-reinforced alternative (Tata Steel, 2016). In 2016, *World Steel* estimated between 3 and 4.5 MT less GHG emissions from a vehicle manufactured from AHSS over conventional steel (WSA, 2020a). Today, nearly all new vehicle designs contain some fraction of AHSS, approaching as much as 60% of the body structure. Recent work which characterizes changes in materials use due to vehicle electrification suggests a four-fold increase in V wt.% from 0.003 for a sedan based on an internal combustion engine (ICE) up to 0.012 for a plug-in hybrid electric (PHEV) SUV (Bhuwalka et al., 2021). With the increasing prevalence of electric (EV) and hybrid electric vehicles (HEV) capable of running zero tailpipe emissions, the importance of the carbon emissions during the production and recycling life phases of the vehicles will become increasingly important and thus the advantages of AHSS over other lightweighting alternatives will only become greater (Hickey, 2019; Lesch et al., 2017; Tata Steel, 2018).

As a result of the wide variability in chemical and processing combinations for AHSSs, quantifying the carbon savings resulting from a single alloying element, i.e., vanadium, is complex and beyond the scope of this work. Furthermore, a detailed model would require access to a complete life cycle inventory comprising proprietary alloy compositions and hundreds of vehicle design parameters (Geyer, 2018; Pero et al., 2018). However, vanadium microalloying plays a critical role in many of the highest performance lightweight steels. The effects of nanoprecipitation strengthening of ferrite in dual-phase steels have been studied extensively (Chakraborti and Mitra, 2007; Garcia et al., 2012; Luo et al., 2010; Son et al., 2005). Here, the inclusion of vanadium results in a simultaneous increase in tensile strength and ductility, two material properties that are ideally sought to be maximized but usually trend inversely (i.e., increased tensile strength typically comes at the expense of ductility). In contrast, alloy additions such as titanium, while providing similar precipitation strengthening effects, yield brittle steels beyond a threshold (*ca.* 0.1 wt.%) (Scott et al., 2013). A similar effect has been documented in TRIP steels where an increase in the ultimate tensile strength (UTS) of up to 200 MPa has been recorded with no loss in elongation toughness upon co-incorporation of vanadium and nitrogen (Oja et al., 2019; Perrard and Scott, 2007; Scott et al., 2004). Indeed, one of the challenges metallurgists face is the ability to increase a material property without the expense of another significantly. For example, as AHSS’ strength rises, so does their susceptibility to hydrogen embrittlement, a form of corrosion wherein the occlusion of diffusible hydrogen embrittles steel, resulting in premature cracking (Cho et al., 2018; Lesch et al., 2017). It has been shown that the inclusion of vanadium serves to trap mobile hydrogen around vanadium carbide inclusions, thereby sequestering hydrogen and reducing the predilection of the material to undergo hydrogen embrittlement.

### Vanadium redox flow batteries

The present study utilizes a base-case (BC) scenario based on the comprehensive life cycle assessment (Weber et al., 2018) of a representative VRFB to calculate the carbon savings for current operational capacity (COC) and the projected operational capacity (POC) under two scenarios (1) *Renewable Energy Curtailment:* where no energy storage technology is in place and (2) *Comparative Assessment*: relative to a lithium-ion battery (LiB) comprised of a lithium-iron-phosphate cathode and lithium titanate anode (Weber et al., 2018).

#### Base case scenario

**Figure 3:**
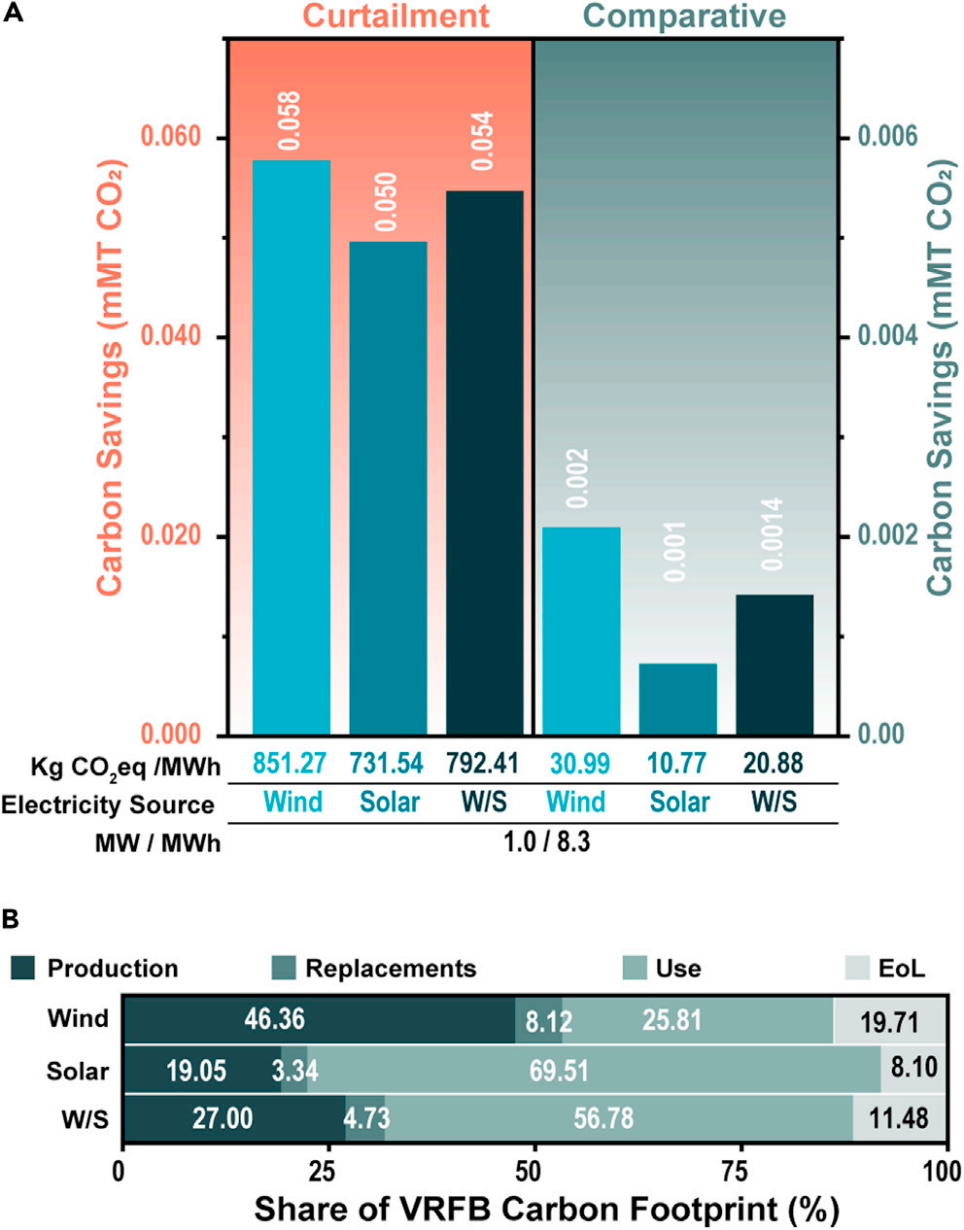
VRFB savings by the base case scenario (A) Savings by the VRFB over a 20-year lifetime are shown in (A) for the curtailment (orange) and comparative assessment (green). The tabulated insets below the column chart are matched to the above columns and indicate (from top to bottom) the normalized savings in kg CO_2_eq/MWh, the colocalized electricity source (wind, solar, or a wind/solar mix (W/S), differentiated by shades of blue), and the rated power and storage capacity of the considered system. (B) The fractional share of each life cycle phase to the total VRFB carbon footprint is shown in (B).

The base case scenario is represented by the VRFB battery considered by Weber et al. with a rated power of 1 MW and a storage capacity of 8.3 MWh (i.e., 1 MW discharged over 8.3 h) (Weber et al., 2018). Both the VRFB and LiB are utilized at an average rate of 1.12 cycles/day over a 20-year lifetime totaling 8,176 charge–discharge cycles and a lifetime provision of 67,861 MWh of electricity (Weber et al., 2018). Carbon savings for the base-case scenario have been calculated assuming a colocalized wind, solar, or 50/50 wind/solar source. A graphic depiction of the 20-year lifetime savings under support from each of the considered electricity sources is provided in Figure 3.

When coupled to a wind source, our analysis suggests that the considered 1 MW, 8.3 MWh installation will save up to 0.058 mMT of CO_2_ over its lifetime (20-years) due to a reduction in renewable energy curtailment, which is equivalent to *ca.* 29,000 MT of coal burned (EPA, 2021). It is important to note that due to the proportion of current overgeneration by renewable sources and limited currently installed energy storage, we have assumed that the total capacity of a VRFB is utilized according to its user profile (i.e., best-case-scenario). Moreover, the details of reduction in emissions enabled by storage depend on multiple factors such as the extent to which the availability of storage increases investments in renewable technologies, regional caps on emissions, subsidies for renewable energy technologies, alternative options for dispatchable sources, and matching of storage to local climate and renewable energy infrastructure (Bistline and Young, 2020; Cavicchi and Ross, 2020). Nevertheless, the potential for renewable energy storage technologies to reduce CO_2_ emissions and renewable curtailment in applications where no grid-energy storage solutions are in place has been demonstrated (Arbabzadeh et al., 2019; Denholm, 2012). For example, Arbabzadehl et al. showed that the coupling of energy storage with pre-existing renewable electricity sources could increase CO_2_ savings from 72% to 90% while reducing renewable energy curtailment from nearly one-third to 9% in California (Arbabzadeh et al., 2019). As the cost of renewable energy storage decreases, the incentive to exploit renewable energy resources more effectively, thus reducing curtailment, increases significantly. Although the carbon costs of unused renewable energy are not high, when demand is greater than supply, increased reliance on energy from non-renewable sources (such as peaker plants) will inevitably spike CO_2_ emissions, thereby significantly offsetting benefits accrued during periods when the renewable energy source is online.

While the benefits of implementing grid-level energy storage are clear, the incentive for one technology versus another has been explored in less detail from a carbon emissions perspective. Weber et al. demonstrated the potential for avoided carbon burden when a VRFB is coupled to a renewable energy source such as wind or solar in place of a LiB (Weber et al., 2018). Their findings estimate that VRFBs produce *ca*. 31 kg CO_2_eq less than a LiB for every MWh of electricity produced by the battery when coupled to a wind source and *ca.* 11 kg CO_2_eq/MWh, when connected to a solar source as reflected by the normalized savings in Figure 3A (these savings consider the benefits of using recycled materials for both the LiB and VRFB). While small compared to the curtailment analysis, the total carbon savings (assuming wind coupling) by a 1 MW, 8.3 MWh VRFB installations total *ca*. 0.0021 mMT of CO_2_, which is equivalent to removing 457 (ICE) passenger cars for one year. The primary origin of savings by a VRFB over a LiB stems from the unsurpassed recyclability of the critical vanadium electrolyte. For instance, a leading producer of high-purity vanadium products has recently demonstrated an unprecedented 97% recovery rate for vanadium electrolytes (U.S. Vanadium, 2021).

The decrease in savings from wind to solar coupling shown in Figure 3A results from the lower round-trip efficiency of the VRFB (75%) compared to a LiB (90%). Here, lower efficiencies lead to excess energy consumption and thus an environmental burden with a magnitude proportional to the carbon intensity of the electricity source. To better illustrate this concept, the share of each of the considered phases to the total carbon footprint of the VRFB is shown in Figure 3B. From wind to solar coupling, the percentage of the use phase increases from 25.81% to 69.51%. Essentially, the carbon costs of the extra energy required to make up for storage inefficiencies are more pronounced for a solar source (106 kg CO_2_eq/MWh) than a wind source (16 kg CO_2_eq/MWh). Accordingly, the kg CO_2_eq saved for every 1 MWh of electricity delivered by the VRFB decreases with an increasing carbon intensity of the coupled electricity source (tabulated insets, Figure 3A). Notably, the LiB technology has the advantage of incumbency – expected improvements in VRFB round-trip efficiency (see Projected operational capacity) coupled with the ongoing grid decarbonization would inevitably drive operating costs down, making the incentive for VRFB selection greater.

#### Current operational capacity

**Figure 4:**
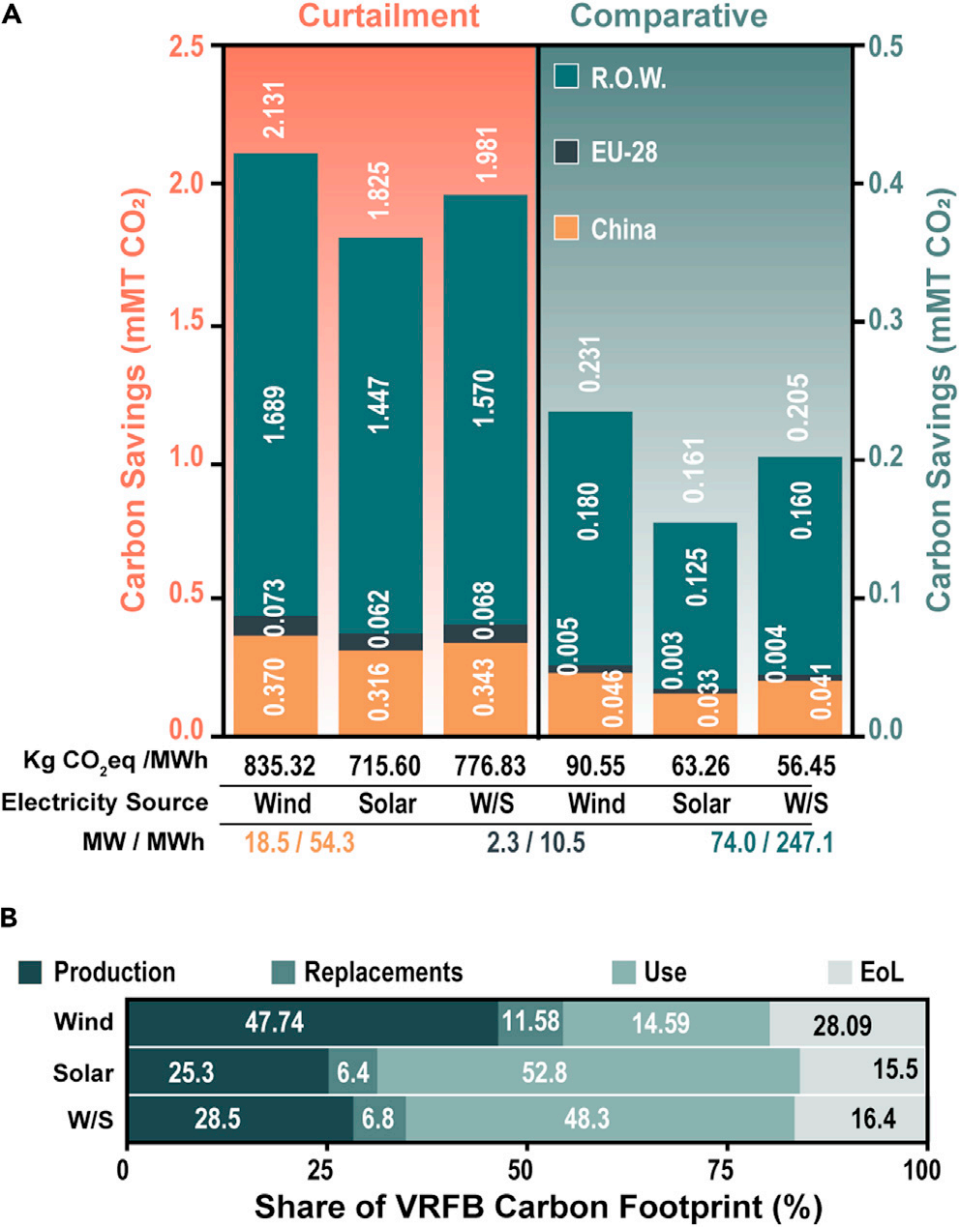
Savings by the current operational VRFB capacity (A) Shows the 20-year savings by all currently operational VRFBs for the curtailment (orange) and comparative assessment (green). Regional savings for China (yellow), EU-28 (black), and R.O.W. (dark green) are stacked to show the total global savings. The tabulated insets below the column chart are matched to the above columns. The normalized savings in kg CO_2_eq/MWh are shown for global data. The colocalized electricity source (wind, solar, or a wind/solar mix (W/S)), and the rated power and storage capacity of the considered region (color matched according to its corresponding column color) are also shown. (B) The fractional share of each life cycle phase to the total VRFB carbon footprint is illustrated in (B).

Based on the carbon footprint of the VRFB and LiB from the BC scenario, CO_2_ savings have been estimated utilizing an extensive compendium of every operational VRFB installation worldwide obtained from Vanitec (see Data S1 for the spreadsheet containing a detailed inventory of every announced, contracted, and operational VRFB project and its geographic location as tabulated by Vanitec from public data sources). While every effort has been made to ensure accuracy in these numbers, deviations are expected where public information is not available. Moreover, it is worth noting that these numbers are likely an underrepresentation of the total operational VRFB installations worldwide, and therefore the savings are a conservative estimate. Like the BC scenario, we assume that the average round-trip efficiency for the VRFB and the LiB is 75% and 90%, respectively, and that each energy storage system is utilized at an average of 1.12 cycles/day over its 20-year lifetime. Much like for the rebar and steel analyses, regional savings by VRFBs are shown for Global, China, EU-28, and R.O.W. regions under the support of a wind, solar, or wind/solar source in Figure 4A.

As discussed in the BC scenario, the carbon intensity of the electricity source has a significant effect on the magnitude of the savings in both the curtailment and comparative assessments. In the interest of clarity of the results and discussion, savings in absolute terms (mMT CO_2_) and the functional unit (kg CO_2_eq/MWh) for the COC are subsequently discussed for a wind-coupled scenario only. Our analysis estimates that current global operational VRFB installations are projected to save up to 2.131 mMT of CO_2_ over their projected 20-year lifetime. To place these savings into perspective, 2.131 mMT of CO_2_ is equivalent to running an additional 413 wind turbines for an entire year. Moreover, annual global carbon savings amount to 0.107 mMT of CO_2,_ which equates to the savings expected from *ca.* 130,000 acres of US forests during the same period (EPA, 2019). The 20-year lifetime savings estimated for China, EU-28, and R.O.W. are 0.370, 0.073, and 1.689 mMT of CO_2_, respectively, and closely follow the installed capacity for each region (insets, Figure 4A).

The tabulated insets in Figure 4A show the normalized global savings in kg of CO_2_eq/MWh delivered by the VRFBs over their lifetime. Compared to the BC scenario, the normalized savings from a reduction of renewable energy curtailment are smaller, i.e., 851.27 and 835.32 kg CO_2_eq/MWh for the BC and COC scenarios, respectively. The decrease in normalized savings from the BC to the COC scenario can be explained by the difference in the average energy-to-power (E/P) ratios – 8.3 for the baseline scenario and 3.3 for the current operational capacity analysis. Here, slower discharge rates (larger E/P ratios) maximize storage capacity, which directly relates to the fraction of renewable energy curtailment (and its carbon costs) avoided by energy storage.

The comparative assessment suggests that selecting a VRFB over a LiB for the currently installed VRFB operational capacity will lead to 0.231 mMT of CO_2_ saved over a 20-year lifetime. It is important to reiterate that while the savings reported in the comparative assessment are an order of magnitude smaller than the curtailment scenario, the comparative assessment is between two green energy storage solutions. In addition to the carbon savings expected from a reduction in renewable energy curtailment, VRFBs stand to save the equivalence of carbon generated from burning 120,000 metric tons of coal over a LiB alternative. In contrast to the curtailment scenario, the lower E/P ratio (faster discharge times) recorded for the COC relative to the BC scenario increases the normalized savings from *ca*. 31 kg CO_2_eq/MWh to *ca*. 91 kg CO_2_eq/MWh. Given that the primary origin of savings by the VRFB over the LiB stems from the production, replacement, and EoL phases (primarily scaled by power in this analysis), a smaller E/P ratio maximizes the lifecycle stages that benefit the VRFB while minimizing the impacts of its lower round-trip efficiency (captured by the use phase which scales with MWh and is shown in Figure 4B). It is worth noting a mixed agreement when comparing the results from a change in E/P ratio with existing literature, in part due to how different E/P ratios are accounted for (Baumann et al., 2017; Hiremath et al., 2015; Rydh, 1999; Weber et al., 2018) (see Limitations of the study for a more detailed discussion). The annualized savings in mMT of CO_2_ are shown for the curtailment and comparative assessment in Table 2.

**Table 2:** Annualized savings by VRFBs from the curtailment and comparative assessment for current operational VRFB capacity.

	Annualized savings (mMT CO_2_)							
	Curtailment				Comparative			
	China	EU-28	R.O.W.	Global	China	EU-28	R.O.W.	Global
Wind	0.0185	0.0036	0.0844	0.1065	0.0023	0.0003	0.0090	0.0115
Solar	0.0158	0.0031	0.0723	0.0913	0.0016	0.0002	0.0063	0.0081
Wind/Solar	0.0172	0.0034	0.0785	0.0990	0.0021	0.0002	0.0080	0.0103

Region-specific savings upon coupling to wind, solar, and a wind/solar mixture have been shown for China, EU-28, R.O.W., and Global regions. To capture each phase of the life cycle proportionately, the one-year savings reported here represent the average yearly savings over the 20-year lifetime, rather than the quantity of savings after 1 year of use.

#### Projected operational capacity

VRFBs, while still a nascent technology, are amid a steep trajectory of growth; several GWh of projects have been announced in recent years. Even if VRFBs only make up a fraction of the impending renewable energy transition, the expected carbon savings from VRFB installations will continue to increase at a sharp pace. Based on the inventory detailed in Data S1, we now consider every VRFB currently operational, under repair, under construction, and announced with a projected operational status by 2030. We estimate that nearly 200 VRFB installations totaling 615 MW of power and 2,485 MWh of storage capacity will be operational by 2030. Once again, this number likely represents a conservative estimate but is nevertheless nearly 800% larger than the COC. Decommissioned installations have been removed from our analyses, whereas publicly announced VRFB installations have been retained. Rather than reporting the expected savings under the support from different renewable electricity sources, we now consider CO_2_ savings from the total number of projected installations—first, assuming the BC scenario round-trip efficiency and cycles/day, next under a scenario where lifetime-cycles are increased from 8,176 to 10,001, and finally under a scenario where the round trip efficiency of the VRFB is improved from 75% to 83% (da Silva Lima et al., 2021). All calculations assume a colocalized electricity source comprised of 50% wind generation and 50% solar generation (60 kg CO_2_eq/MWh).

**Figure 5:**
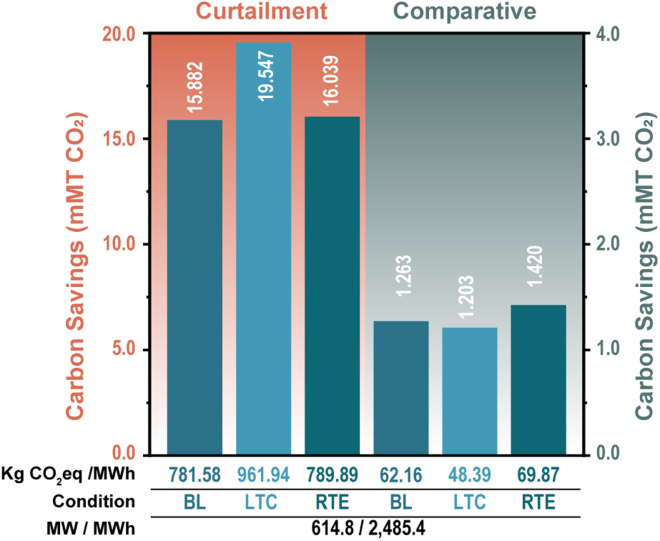
Savings by the projected VRFB operational capacity The estimated lifetime savings for the projected operational capacity of VRFBs are shown for the curtailment (orange) and comparative assessment (green). The tabulated insets below the column chart are matched to the above columns and indicate (from top to bottom) the normalized savings in kg CO_2_eq/MWh, the condition (baseline (BL), an increase in lifetime cycles (LTC), or an improvement in round trip efficiency (RTE)) – differentiated by shades of blue, and the projected operational power and storage capacity.

The projected 20-year savings from the baseline scenario (8176-lifetime cycles, 75% round trip efficiency) as a result of reduction of renewables curtailment amount to 15.88 mMT of CO_2_, and are equivalent to avoiding nearly 8 mMT of coal burned and the savings expected from recycling 5.4 mMT of waste. Furthermore, relative to LiBs, VRFBs could save as much as 1.26 mMT of CO_2_. Figure 5 shows that an increase in the number of lifetime cycles drives the savings from 15.88 mMT to 19.55 mMT of CO_2_; the origin of these increased savings is relatively straightforward–an increase in lifetime cycles leads to a more significant reduction of curtailed renewables and thus an increase in the expected carbon savings. Owing to the lower round-trip efficiency of VRFBs, an increase in the use phase relative to the mass components favors LiBs (in this calculation), but VRFBs remain the less carbon-intensive option. However, it is important to note that, unlike a LiB, the number of charge–discharge cycles expected from a VRFB can be increased significantly without the need for replacement/maintenance. This benefit has not been considered here but is worth exploring, in more detail, in future work. As shown in Figure 5, an increase in the round-trip efficiency of VRFBs from 75% to 83% increases the normalized savings recorded for both the curtailment and comparative assessments.

The massive savings estimated from the curtailment analysis suggests that at the current state of the energy transition, considerable savings in carbon emissions are possible even after debiting the carbon costs of producing, operating, maintaining, and recycling the energy storage infrastructure. Even in their nascency, VRFBs show a smaller carbon footprint than a LiB when coupled to a renewable source resulting from the disproportionate recyclability of a VRFB and LiB. Ongoing efforts to lower the carbon impacts associated with vanadium production will inevitably reduce the carbon costs during the production of first-generation installations which utilize virgin vanadium. Similarly, technological improvements enabling greater VRFB round-trip efficiency and ongoing efforts to decarbonize electricity production will drive the carbon costs of their operation down.

### Conclusions

The race to limit global warming to no more than 1.5°C as articulated in the Paris Climate Agreement will require a sustained effort spanning decarbonization of hard-to-abate industrial sectors coupled with a transition to renewable sources of energy. Widespread changes in industrial processes, deep decarbonization of manufacturing economies, and the energy transition will set in motion intense competition for natural resources, whose sustainable utilization will be critical to economic prosperity and global plans for mitigation of climate change. A wide range of clean energy technologies is enabled by materials that face current or emerging criticality concerns. As the energy transition gathers pace, the demand for these critical raw materials, which are crucial to the energy transition, will grow, and so will the importance of supply chain resilience, recycling, and policy decisions to address the sustainable sourcing and utilization of these minerals. In this work, we have utilized industry data on the consumption of vanadium in different sectors and compilations of public data on large-scale grid-level storage to develop a detailed assessment of the potential impact of vanadium on decarbonizing the construction industry and enabling the energy transition. Based on the sum of the estimated savings from steel sections (1.18 mMT of CO_2_) and the average values between the lower and upper bounds of the rebar (183.5 mMT of CO_2_) and VRFB COC analyses (0.0989 mMT of CO_2_), we estimate that approximately 185 mMT of CO_2_ are avoided annually from the use of vanadium in the construction and energy storage sectors. To place these savings in perspective, 185 mMT of CO_2_ equates to nearly 93 mMT of coal burned or *ca.* 430 million barrels of oil consumed. Notably, these savings have originated from both the hard-to-abate sectors and nascent green energy technologies alike, demonstrating the role of vanadium in improving the energy efficiency of heavy industries while enabling the energy transition through the provision of energy storage to balance the intermittency of renewable energy sources (IEA, 2020b). It is worth noting that CO_2_ savings from the automotive industry, while discussed qualitatively in this work, have not been quantified and likely represent a significant addition to the total savings made possible by vanadium products. The versatility of vanadium and its ability to create carbon savings within multiple sectors highlight the importance of applying a life cycle assessment to policy decisions. The study further underscores the vulnerability of essential decarbonization and energy transition to supply chain disruptions and price volatility, suggesting the need for extensive emphasis on recycling and a substantial expansion of primary vanadium extraction. With the expected growth of grid-level energy storage, given the clear benefits of VRFBs over not having storage options or alternative short-term storage technologies, an increasing proportion of vanadium demand will be driven by this technology, further driving the need for vanadium production beyond closed-loop processes operational in the steel industry.

## Limitations of the study

One of the main limitations of the steel LCA approach is that the ICE database was developed in 2008 and revised in 2011 and, therefore, may be susceptible to temporal variations. Furthermore, this database is prepared using global and European averages for energy values, and mainly UK sources were used for the carbon values, thus having a limited geographic representation (Hammond and Jones, 2008). The vanadium data is relatively recent (2019) and, therefore, less prone to temporal variations. Given the limited availability of data associated with rebar consumption in the EU-28, the upper bounds of the rebar analysis are subject to a greater level of uncertainty relative to the lower bounds, which are calculated utilizing comprehensive China and global rebar data.

An existing limitation for the VRFB analyses is that the current operational capacity and projected operational capacity scenarios do not fully account for the effects of energy density when comparing distinctive E/P configurations – energy storage systems with higher energy densities (such as LiBs) often require fewer cells to provide similar storage capacity. In addition, unlike most other energy storage technologies (including LiBs), VRFBs can be designed flexibly according to the energy and power requirements of the application. In other words, during production, the mass components that contribute to the capacity (MWh) of a VRFB (i.e., electrolyte) can be scaled independently from those that determine its power rating (MW) (i.e., stack components). Weber et al. show that on a mass basis, the power components of a VRFB show greater environmental impact than the vanadium electrolyte, suggesting that the carbon footprint of a VRFB is minimized for high E/P ratios per MWh of storage capacity (Weber et al., 2018). As noted above, the use-phase is critical to the carbon emissions discussed in terms of the functional unit, kg CO_2_eq/MWh provided by the battery over the lifetime. While higher E/P ratios may minimize the carbon impact per MWh of storage capacity, under the same use profile (i.e., number-of-cycles per day) a VRFB with a higher E/P ratio will deliver greater MWh over its lifetime, increasing the relative GWP contribution of the use phase. One approach, similar to the one utilized in this work, accounts for the dependency of storage capacity and power output on the carbon impacts of a VRFB by applying an exponential relationship between the membrane area (determined by power) and the electrolyte volume (determined by capacity) to establish a scaling factor for the production costs of a representative VRFB (Baumann et al., 2017). Future work will focus on developing updated life cycle inventories that directly evaluate the life cycle impact of specific VRFB installations with different E/P ratios. In summary, the quantification of CO_2_ savings in the comparative assessment of the COC and POC scenarios is variable due to distinct E/P ratios.

## STAR★Methods

### Key resources table

**Table:** 

REAGENT OR RESOURCE	SOURCE	IDENTIFIER
**Deposited Data**		

Inventory of Carbon & Energy (ICE)	Hammond and Jones (2008)	https://doi.org/10.1680/ener.2008.161.2.87
Structural and machine learning models	Pradeep Kumar et al. (2021)	https://doi.org/10.1039/D0EM00424C
China steel consumption	Santos et al. (2021)	https://doi.org/10.1016/j.matt.2020.12.009
Compendium of vanadium redox flow battery installations	This work	https://doi.org/10.1016/j.isci.2021.103277
Estimated CO_2_ emissions by region	Global Carbon Project	https://www.globalcarbonproject.org/

**Software and algorithms**		

ETABS	Computers and Structures, Inc.	https://www.csiamerica.com/products/etabs
Revit	Autodesk	https://www.autodesk.com/products/revit
Microsoft Excel	Microsoft	https://www.microsoft.com/en-gb/

**Other**		

Greenhouse Gas Equivalencies Calculator	The United States Environmental Protection Agency	https://www.epa.gov/energy/greenhouse-gas-equivalencies-calculator

### Resource availability

#### Lead contact

Further information and requests for resources should be directed to and will be fulfilled by the lead contact, Sarbajit Banerjee (banerjee@chem.tamu.edu)

#### Materials availability

This study did not generate new materials.

### Method details

An original life-cycle assessment has been developed for steel sections according to ISO 14040(ISO 14040, 2006) and ISO 14044 (ISO 14044, 2006) standards (Pradeep Kumar et al., 2021). Savings from the automotive sector have been discussed qualitatively in this work, given the complexity of proprietary alloy compositions and limited data on volumes of different alloys used in specific automotive models. Extrapolated savings for VRFBs have been normalized for the chosen functional unit in this work and are based on a previously published life cycle assessment (Weber et al., 2018) of a representative VRFB that meets ISO 14040 (ISO 14040, 2006) and ISO 14044 (ISO 14044, 2006) standards. While not highlighted here, it is worth noting that other uses of vanadium include as the primary catalyst in the production of sulfuric acid by the contact process (Marwa et al., 2017), removal of NO_*x*_ emissions from burning coal, oil, and gas in the power industry and in selective catalytic reduction of NO_*x*_ to treat diesel exhaust emissions (Chemicals Sector, Figure 1); and in Ti-Al-V alloys for aerospace applications (Titanium Sector, Figure 1) – the avoided environmental burden from these sectors has not been considered further in this work as a result of the low substitution potential of vanadium and the absence of credible alternatives in these sectors.

#### Construction sector: structural steel sections and reinforcement bars

The construction industry accounts for over 50% of the global steel demand, of which 44% is used in the production of reinforcing bars, 25% for structural steel sections, and 31% for sheet products(WSA, 2020b). A life cycle assessment is carried out using 2019 market data related to the consumption of steel and vanadium in the construction industry. The following sections detail the methodology applied in this study, adopted from our previous work on vanadium-microalloyed steel reinforcing bars (Pradeep Kumar et al., 2021).

##### Structural and machine learning models

The primary purpose of the structural model is to determine the quantities of material required by different grades of steel sections to achieve the same load-carrying capacity. Grade S235 (235 MPa ∼ 34 ksi) steel (mild steel) is taken as the reference case for the analysis and is compared with a higher strength grade S350 (350 MPa ∼ 50 ksi) steel. The modeling framework is divided into two levels: component and building-level analysis. To perform the structural analysis and modeling, the following structural design standards are used: (a) EN 1990: structural design details(EN 1990:2002+A1, 2005); (b) EN 1991-1-1: dead and live load specifications for buildings(EN 1991-1-1, 2002); (c) EN 1991-1-4: wind load specifications(EN 1991-1-4, 2010); (d) EN 1993-1-1: all analysis and design parameters for steel structures(EN 1993-1-1, 2005); (e) EN 10025-2:2019 (E): mechanical properties of steel (EN 10025-2: 2019 E, 2019). An example procedure published by The Steel Construction Institute serves as a model for the component level analysis (Brettle, 2009).

For the component level model, structural steel framing components such as steel beams and columns are analyzed based on the procedure delineated by the steel building design report (Brettle, 2009). Figures S2A and S2B show the 3D rendition of steel beam and column sections. Specifications for the analysis and design parameters conform to Eurocode 3 (EN 1993-1-1, 2005). The quantity of steel required by sections with different yield strengths is computed and compared to determine material savings. To perform a building level structural analysis, a four-story hypothetical building is modeled as shown in Figure S2C using ETABS v18 structural software (Pradeep Kumar et al., 2021). The building has 5 bays (each 7 m long) × 3 bays (5.5 m) with a story height of 4 m. Roof type H and building category C1 as per EN 1991-1-1 (EN 1991-1-1, 2002) are adopted for the model. Dead, live, and wind loading parameters conforming to EN 1991-1-1 (EN 1991-1-1, 2002) and EN 1991-1-4 (EN 1991-1-4, 2010) are used. The beams and columns are modeled as structural steel sections. I-sections are used for beams, whereas hollow box sections are used for columns. Eurocode 3 (EN 1993-1-1) (EN 1993-1-1, 2005) is used to perform the analysis and design. For floor slabs, composite steel decking is used following SCI P300 (Rackham et al., 2014). For both component and building-level models, grade S350 with a yield strength of 350 MPa (∼50 ksi) is used as the vanadium-microalloyed steel grade and is compared with mild steel (grade S235, sans vanadium) with a yield strength of 235 MPa (∼34 ksi). Vanadium weight percentages corresponding to each of the considered steel grades were calculated from a previously detailed machine learning model to arrive at an average value that is representative of aggregated data and not specific to a single trial (Pradeep Kumar et al., 2021)*.*

Structural analysis and design of steel sections are performed to obtain the quantities of steel required to meet a set of structural specifications for a given steel grade. The vanadium weight percent values are derived from the machine learning model and subsequently combined with structural modeling results to calculate steel savings, which are translated to embodied energy and carbon savings. The steel beams are analyzed and designed at the component level for bending resistance, lateral-torsional buckling resistance, shear resistance, and service limit state. For the assumed loading conditions, universal beams (UB) according to BS4 Part 1 1993 (BS4 Part1, 1993) are considered for design. The steel columns are analyzed and designed for flexural buckling resistance, lateral-torsional buckling resistance, and combined bending and axial compression buckling. Universal Columns (UC), according to BS4 Part 1 1993 (BS4 Part1, 1993), are considered for the design under the assumed loading condition. At the building level, a maximum slenderness ratio is specified (according to EN 1992-1-1) for the beams and columns to avoid buckling, thereby limiting the amount of cement reduction in a load-bearing application (Pradeep Kumar et al., 2021).

##### Life cycle assessment (LCA)

*System Boundary*: A cradle-to-site gate system boundary is considered for the LCA, which covers the production of building materials and their transportation to the construction site. The material production stage includes extraction of raw materials, processing of raw materials, manufacturing, finishing, packaging, storage, and all transportation involved within these activities.

*Functional Unit*: Following the ISO 14040 (ISO 14040, 2006) and 14044 (ISO 14044, 2006) recommendations, the two functional units considered include MJ/m^3^ and kg CO_2_eq/m^3^ for embodied energy and carbon, respectively. These functional units are used for performing LCA; however, the regional level savings in embodied energy and carbon emissions are presented in absolute units based on available market data.

*Impact Assessment*: To perform the LCA, first, the Inventory of Carbon and Energy (ICE v2.0) database that reports the embodied energy (MJ/kg) and embodied carbon (kg CO_2_eq/kg) for different buildings materials are referred to. Specifically, the embodied energy and carbon values of structural steel sections and rebars for different regions are derived from the ICE database compliant with ISO standards.(Hammond and Jones, 2008) Table S2 lists the energy and carbon values for structural steel sections from ICE v2.0; corresponding values for vanadium were obtained from the literature (Nuss and Eckelman, 2014; Weber et al., 2018). A regional impact assessment is carried out to evaluate the embodied energy and environmental impacts of using vanadium-microalloyed steel. The structural steel quantities obtained from the structural modeling along with the amount of vanadium required to achieve the yield strength (obtained from the machine learning model) are used together with the embodied energy and carbon values of structural steel to compute embodied energy and carbon savings. The embodied carbon savings reported in this paper represent only carbon emissions and do not include other GHG emissions.

#### Automotive sector

Carbon savings resulting from vanadium-based AHSS steels in automotive designs have been only discussed qualitatively in this work.

#### Vanadium redox flow batteries

In this work, we utilize extrapolated data from a previously published life-cycle assessment (Weber et al., 2018) (compliant with ISO 14040 (ISO 14040, 2006) and ISO 14044 (ISO 14044, 2006) standards) to demonstrate the benefits, from a carbon savings perspective, of a vanadium redox flow battery (VRFB) under two scenarios: (1) *Renewable Energy Curtailment:* where no energy storage technology is in place and (2) *Comparative Assessment*: relative to a lithium-ion battery (LiB) comprised of a lithium-iron-phosphate cathode and lithium titanate anode. CO_2_ savings have been calculated in absolute terms for a base case (BC) scenario, current operational capacity (COC), and the projected operational capacity (POC) by 2030.

The functional unit in this work is CO_2_eq/1 MWh of electricity delivered by the battery over a 20-year lifetime. Unlike steel, whose savings are fully realized after production, savings by a VRFB continue during its lifetime Therefore, to demonstrate total carbon savings over the lifetime of the VRFB, CO_2_ savings have been calculated in absolute terms for a 20-year lifetime under support from renewable energy sources, i.e., wind and solar. For the COC analysis, annual savings are reported to establish the normalized unit utilized in this work for China, EU-28, and R.O.W. installations – to capture each phase of the life cycle proportionately, the one-year savings reported here derive from the average yearly savings over the 20-year lifetime, not the quantity of savings after one year of use.

##### Renewable energy curtailment

At the current stage of the energy transition, the quantity of overgeneration across most of the world is much larger than the total capacity of installed grid-level energy storage (Arbabzadeh et al., 2019; Canbulat et al., 2021; O’Shaughnessy et al., 2020); as such, a reasonable assumption is that currently, the full capacity of a VRFB will be utilized as per its specified use profile, i.e., number-of-cycles/day. To calculate the carbon savings made possible by a VRFB in a scenario where no other storage options are in place, we assume that the total renewable electricity that a battery could have stored must be later generated by a dispatchable source of electricity such as coal, according to Equation 1:

(Equation 1) $$Carbon\;Savings=C_{Coal}-\left(C_{Renewable}+C_{VRFB}\right)$$

where $C_{Coal}$ denotes the carbon footprint of the total electricity production from a coal source (888 kg CO_2_eq/MWh delivered) (Report, 2011), $C_{Renewable}$ represents the carbon footprint of producing the same quantity of electricity from a renewable source (wind or solar, 16 kg CO_2_eq/MWh, and 106 kg CO_2_eq/MWh, respectively)(Report, 2011; Weber et al., 2018), and $C_{VRFB}$ represents the total carbon footprint of the VRFB (Weber et al., 2018). Notably $C_{VRFB}$ accounts for the carbon penalty incurred by electricity loss due to VRFB round-trip inefficiencies (*vide infra*). While not insignificant, the additional carbon savings resulting from a reduction of transmission and distribution losses have not been accounted for here, but it is worth noting that they likely offset, to an extent, the costs of electricity losses due to round-trip inefficiencies (Jewell and Hu, 2012).

##### Comparative assessment

Several life cycle assessments have been reported for both LiBs and VRFBs; however, variability in system boundaries, user profiles, and functional units has led to a large range in the reported carbon footprint of each technology (Baumann et al., 2017; da Silva Lima et al., 2021; Hiremath et al., 2015; Weber et al., 2018). The work by Weber et al. represents a noticeable exception that applies the same rigor to a life cycle assessment of a typical VRFB and LiB. A comprehensive life cycle inventory is available from the original work and forms the basis for the calculations presented here (Weber et al., 2018). For each calculation, each storage system’s production, replacement, use, and end-of-life (EoL) phases have been considered. For the production phase, the benefits of recycling have been accounted for by the avoided burden method, which considers the use of recycled materials from a previous application to be free of the environmental burden during subsequent applications (Nordelöf et al., 2019). In contrast to LiBs, VRFBs can be designed according to the energy and power requirements of the use case; here, the number of cells (stack and periphery components) can be increased to optimize power (MW), whereas the quantity of vanadium electrolyte can be increased to maximize storage capacity (MWh) (Baumann et al., 2017). Accordingly, the environmental impacts associated with electrolyte, stack, and periphery components have been scaled independently to account for variability in the discharge period across all the considered scenarios i.e., the mass fraction of the electrolyte, stack, and periphery components (each with their distinct carbon footprint) will vary between similarly sized installations that have been optimized for purposes of either frequency regulation or peak support (Baumann et al., 2017; Hiremath et al., 2015; Weber et al., 2018).

A 75% and 90% round-trip efficiency from the VRFB and LiB, respectively, is maintained for the BC scenario and COC analysis. In other words, to deliver 1 MWh, approximately 1.33 MWh, and 1.11 MWh must be charged by the electricity source for the VRFB and the LiB, respectively—here the carbon costs that originate from the excess 0.33 MWh and 0.11 MWh generated are attributed to the use phase of the VRFB and LiB, respectively (Weber et al., 2018). For the COC and POC scenarios, the carbon impacts from each stage are scaled independently, utilizing the carbon costs from each phase of the BC scenario. For the VRFB, stack production, replacements, and the end-of-life phases were scaled against installed power (MW), whereas electrolyte production and use-phase were scaled according to energy capacity (MWh). Since Li-ion batteries cannot be dimensioned separately for power rating and energy capacity, the entire production phase has been scaled by the rated power and the use phase was scaled according to the rated capacity of the COC and POC. For a discussion of the limitations to this approach, see the Limitations of this study section.

## Data Availability

• All data reported in this paper will be shared by the lead contact upon request. • This paper does not report original code. • Any additional information required to reanalyze the data reported in this paper is available from the lead contact upon request.

## Supplemental information

Document S1. Figures S1 and S2 and Tables S1 and S2

Data S1. Compendium of vanadium redox flow battery installations (2019), related to Figures 3–5
