# Supplementary material for: Assessing the role of vanadium technologies in decarbonizing hard-to-abate sectors and enabling the energy transition
Source: iScience. 2021 Oct 13;24(11):103277. doi: 10.1016/j.isci.2021.103277 (PMC8564109; doi:10.1016/j.isci.2021.103277)
Supplement: Document S1. Figures S1 and S2 and Tables S1 and S2 [file mmc1.pdf]

iScience, Volume *24*

Supplemental information

Assessing the role of vanadium technologies in decarbonizing hard-to-abate sectors and enabling the energy transition

David A. Santos, Manish K. Dixit, Pranav Pradeep Kumar, and Sarbajit Banerjee

Table S1. Results of embodied energy and carbon savings, related to Table 1.

Savings for the component level- and building level- analyses comparing V steel grades to mild steel. Embodied energy and carbon values are obtained using EU-28 EE and EC values (listed in Table S2). EE: embodied energy; EC: embodied carbon (yield strength shown in bold denotes mild steel as the baseline)

Yield Strength (MPa)	Component Level				Building Level	
	Steel Beam		Steel Column			
	% Savings				%Savings	
	EE	EC	EE	EC	EE	EC
**235**	0.00%	0.00%	0.00%	0.00%	0.00%	0.00%
**350**	10.04%	10.11%	39.10%	39.15%	5.28%	5.36%

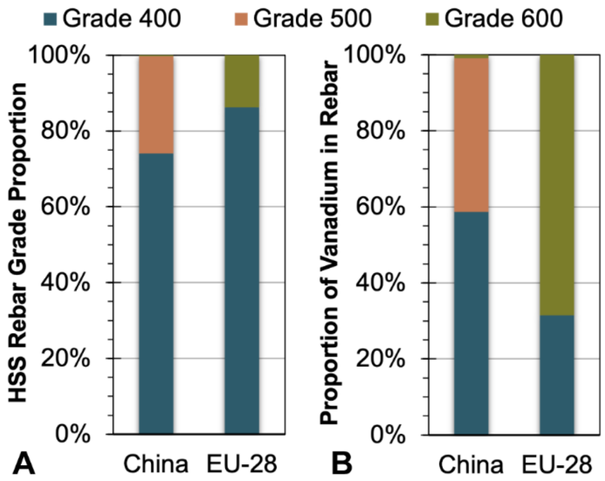

Figure S1. Proportion of different grades of high-strength rebar, related to Figure 2.

(A) High-Strength Steel (HSS) rebar grades (Grade 400 – 400MPa, Grade 500 – 500MPa, Grade 600 – 600MPa) and **(B)** Vanadium in respective HSS rebar grade in 2019 for different regions considered in the study

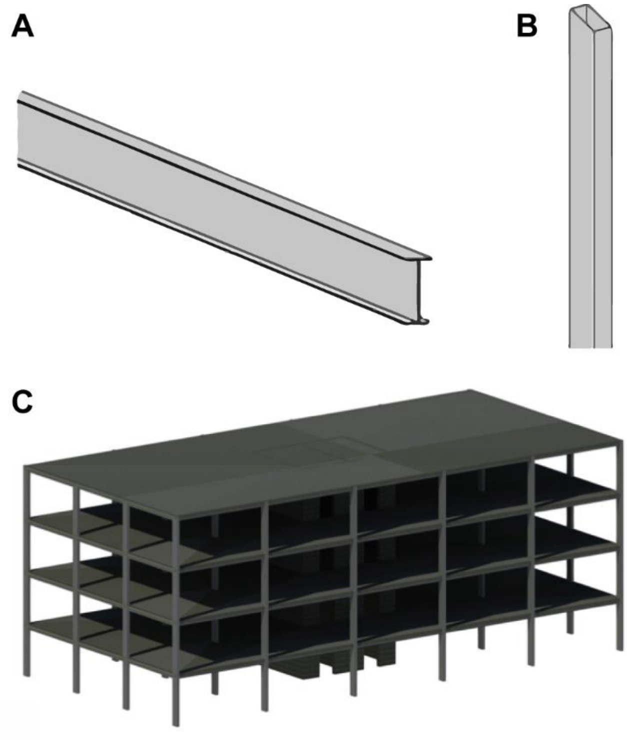

Figure S2. 3D Rendition of the structural steel sections and hypothetical building, related to STAR Methods.
(A) Structural steel beam (I-) section; **(B)** Structural steel column (hollow) section; and **(C)** rendition of the hypothetical building model with structural steel beam and column sections as sketched using Revit

Table S2. Carbon and energy data for structural section steel, rebar steel, and vanadium, related to STAR Methods.
The value for steel is extracted from ICE v2.0 – UK typical with EU 27 3-year average recycled content of 59% in steel production for EU-28, rest of the world (R.O.W.) typical with 3-year average ROTW recycled content of 35.5% in steel production for R.O.W. and China, and world typical with 3-year world average recycled content of 39% in steel production for the entire world.

	**Region**	**Embodied Energy (MJ/ kg)**	**Embodied Carbon (kg CO_2eq/ kg)**
	EU-28	21.5[54]	1.53[54]
**Section Steel**	R.O.W./China	28.1[54]	2.12[54]
	World	27.1[54]	2.03[54]
	EU-28	17.4[54]	1.40[54]
**Rebar steel**	R.O.W./China	22.3[54]	1.95[54]
	World	21.6[54]	1.86[54]
**Vanadium**	All	516[55]	33.1[55] - 39.1[43]
